# Cellular mechanisms of hormone secretion in neuroendocrine tumors: what goes wrong?

**DOI:** 10.3389/fcell.2025.1527083

**Published:** 2025-07-01

**Authors:** Laura Streit, Emeline Tanguy, Laurent Brunaud, Petra Tóth, Nicolas Vitale, Stéphane Ory, Stéphane Gasman

**Affiliations:** ^1^ Centre National de la Recherche Scientifique, Université de Strasbourg, Institut des Neurosciences Cellulaires et Intégratives, Strasbourg, France; ^2^ Département de Chirurgie Viscérale, Métabolique et Cancérologique (CVMC), INSERM NGERE-U1256, Université de Lorraine, CHRU Nancy, Hôpital Brabois adultes, Vandoeuvre-lès-Nancy, France

**Keywords:** neuroendocrine tumors, hormone, secretion, vesicular trafficking, exocytosis, dysregulated genes and proteins

## Abstract

Neuroendocrine tumors (NETs) constitute a heterogeneous group of neoplasms arising from hormone-releasing cells. Secretion of hormones stored in vesicles occurs through calcium-regulated exocytosis, a process that needs to be tightly controlled to avoid unbalanced levels of hormones. A critical feature shared by most of the NETs is a dysfunctional secretory pathway mainly leading to hypersecretion, which often induces clinical complications. In this review, we focus on the cellular process of hormone exocytosis and discuss the potential molecular mechanisms leading to deregulated hormone secretion in various NETs. Particular attention is paid to expression level modifications for genes and proteins involved in the exocytic pathway in NETs.

## 1 Introduction

**FIGURE 1:**
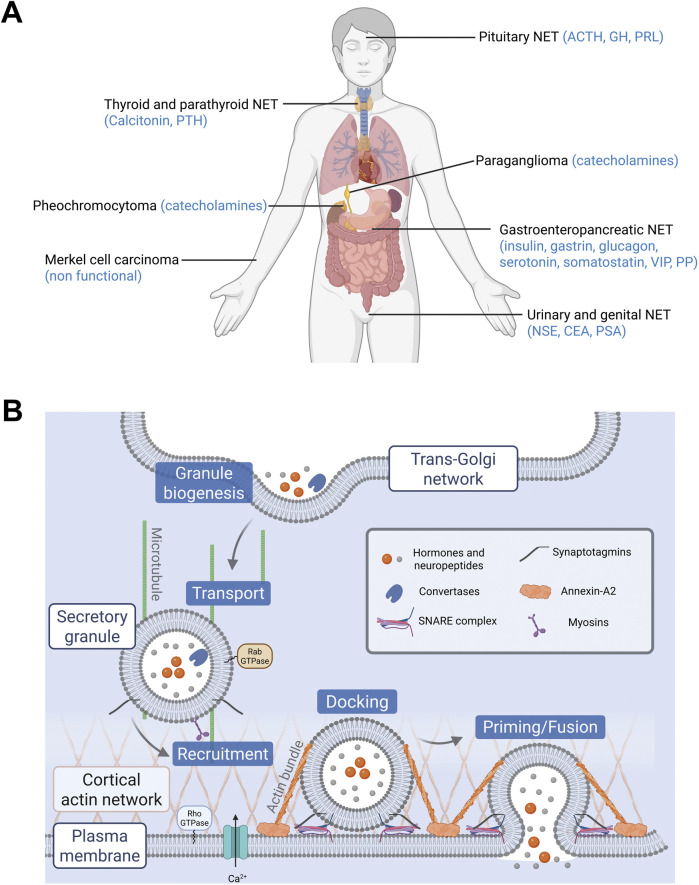
**(A)** Localization of neuroendocrine tumors (NETs) and their main secreted hormones. Hormones abbreviations: ACTH, adrenocorticotropic hormone; CEA, carcinoembryonic antigen; GH, growth hormone; NSE, neuron specific enolase; PRL, prolactin; PP, pancreatic polypeptide; PSA, prostate specific antigen; PTH, parathyroid hormone; VIP, vasoactive intestinal peptide. **(B)** Main steps of exocytosis and key proteins involved. Granules containing hormones and neuropeptides are formed at the trans-Golgi network by budding. They are then transported to the cell periphery and recruited to the plasma membrane. Their docking and priming prepare them for the step of fusion. Finally, the granules release their contents following calcium stimulation.

Neuroendocrine tumors (NETs) are derived from neuroendocrine cells that control pleiotropic physiological functions by releasing various hormones and neuropeptides. NETs constitute a highly heterogeneous group of neoplasms in terms of function and morphology, mainly because neuroendocrine cells are spread all over the body as dispersed cells (diffuse neuroendocrine system) or concentrated in organs (Figure 1A). However, one of the most common physiological features of these NET family members is the dysfunction of hormone and neuropeptide secretion, leading to hypersecretion and eventually to clinical symptoms. For example, acromegaly often results from excessive secretion of growth hormone by pituitary adenoma (Dineen et al., 2017). Hypersecretion of serotonin by carcinoid tumors from the gastro-intestinal tract can trigger carcinoid syndrome, which is associated with flushing, diarrhea, bronchoconstriction and cardiac valvular disease (Onaitis et al., 2000). Excessive levels of circulating catecholamines in patients with pheochromocytoma, a NET derived from the adrenal medulla chromaffin cells, induce hypertension potentially leading to cardiomyopathies and stroke (Zhang et al., 2017; Y-Hassan and Falhammar, 2020). Moreover, enhanced secretory activity of NET cells may develop over time with negative impact on prognosis. For instance, a silent pituitary adenoma may progress into an actively secreting form, while a non-functional pancreatic tumor can start secreting excessive levels of hormones, thus evolving to a more aggressive tumor phenotype (Brown et al., 2006; Daems et al., 2009). In small cell lung cancer (SCLC), a high-grade malignant cancer, the progressive neuroendocrine nature of cancer cells enhances secretion of a variety of neuropeptides, together with growth factors that significantly accelerate the invasive growth by their autocrine action. Dysfunction of hormone secretion in NETs has been known from the clinical point of view for a long time, but the cellular and molecular mechanisms disturbing secretory pathways in tumor cells are rarely explored and remain poorly understood.

The first idea that usually comes in mind is that the increased level of secreted molecules results from an increased number of hormone-secreting cells within the tumor. However, the simplistic view of “the more secreting cells, the more secreted hormones” fails to consider alternative mechanisms. For example, in human pheochromocytoma, we recently reported using carbon fiber amperometry that individual tumor cells release significantly higher amounts of catecholamines compared to non-tumor chromaffin cells (Houy et al., 2022). The obvious conclusion from these observations is that the process of hormone release itself may be dysregulated in NET cells.

Hormone and neuropeptide secretion by neuroendocrine cells occurs through calcium-regulated exocytosis, a process that has been the subject of extensive studies over the past 3 decades (Anantharam and Kreutzberger, 2019). In these cells, hormones are synthesized and then stored in large dense core vesicles (LDCV) also known as secretory granules. Exocytosis is triggered by extracellular stimuli that increase intracellular calcium level and involves several tightly regulated steps including, i) the recruitment of the secretory granules to the cell periphery, ii) their docking to exocytic sites, which physically attaches them to the plasma membrane, iii) their priming, which prepares the granules for fusion, and finally iv) the fusion of the secretory granule membrane with the plasma membrane, leading to the release of luminal content of secretory granules (Figure 1B). The key questions arising from the observation of hypersecretion at the single cell level are which specific steps of the exocytic pathway are dysregulated in NETs and which proteins might be involved in this amplified secretory activity? Most of the limited data available today comes from “omics” approaches, which reveal changes in gene and protein expression across different NETs. These data are primarily from NETs derived from adrenal medulla, thyroid and pituitary gland. In this review, we focus on studies that investigate gene or protein expression changes specifically in human tumor biopsies. From this literature, we summarize the expression of key proteins involved in distinct steps of the hormone secretion pathway. Table 1 lists these main proteins and genes, highlighting their function in exocytosis and their expression changes in NETs. In the following sections, we examine each step of the hormone secretory pathway, from granule formation and cargo loading to vesicle fusion and membrane remodeling, highlighting the alterations observed in human NET samples.

**TABLE 1:** List of up- and downregulated proteins or genes in neuroendocrine tumors.

Gene	Protein	Functions	Uniprot entry	Variation in tumors	References
ANO6	Anoctamin-6	Plasma membrane phospholipid scrambling, lipid reorganization of the plasma membrane	Q4KMQ2	↗ pheochromocytoma vs. non tumor (protein)	Unpublished data
				↘ lactotroph pitNET vs. normal cells (gene)	Cui et al. (2021)
ANXA2	Annexin-A2	Calcium-dependent protein binding, cytoskeleton reorganization, vesicle fusion, vesicle budding from membrane	P07355	↘ pheochromocytoma vs. non tumor (protein)	Houy et al. (2022)
				↗ MTC vs. normal thyroid (protein)	Kurczyk et al. (2020)
				↘ GH-secreting pitNET vs. respective normal pituitary (protein)	Ribeiro-Oliveira et al. (2008)
				↗ somatotroph pitNET vs. normal cells (gene)	Cui et al. (2021)
				↗ parathyroid adenoma vs. normal parathyroid (protein)	Giusti et al. (2011)
ATP8A1	Phospholipid-transporting ATPase IA	Phospholipid flippase, lipid reorganization and control of vesicle trafficking at the plasma membrane	Q9Y2Q0	↗ pheochromocytoma vs. non tumor (protein)	Houy et al. (2022)
				↘ lactotroph pitNET vs. normal cells (gene)	Cui et al. (2021)
ATP9A	Probable phospholipid-transporting ATPase IIA	Phospholipid flippase, lipid reorganization and control of vesicle trafficking at the plasma membrane	O75110	↗ pheochromocytoma vs. non tumor (protein)	Unpublished data
CADPS	Calcium-dependent secretion activator 1	Priming, fusion, calcium binding, plasma membrane binding	Q9ULU8	↗ pheochromocytoma vs. non tumor (protein)	Houy et al. (2022)
				↗ parathyroid adenoma vs. normal parathyroid (protein)	Arya et al. (2019)
CADPS2	Calcium-dependent secretion activator 2	Priming, calcium binding, plasma membrane binding, SNARE binding	Q86UW7	↗ pheochromocytoma vs. non tumor (protein)	Houy et al. (2022)
CHGA	Chromogranin A	Granule biogenesis, secretory granule organization, peptide hormone processing	P10645	↗ pheochromocytoma vs. non tumor (protein)	Houy et al. (2022)
				↗ sporadic MTC vs. paired normal thyroid tissue (protein)	Zhan et al. (2018)
				↗ MTC vs. normal thyroid (protein)	Kurczyk et al. (2020)
				↗ siNENs vs. normal human enterochromaffin cells (gene and protein)	Giovinazzo et al. (2013)
CHGB	Secretogranin-1	Secretory granule biogenesis and organization, peptide hormone processing	P05060	↗ pheochromocytoma vs. non tumor (protein)	Houy et al. (2022)
				↗ sporadic MTC vs. paired normal thyroid tissue (protein)	Zhan et al. (2018)
				↗ somatotroph pitNET vs. normal cells (gene)	Cui et al. (2021)
CPLX2	Complexin-2	Positively regulates a late step in exocytosis of various cytoplasmic vesicles, such as synaptic vesicles and other secretory vesicles	Q6PUV4	↗ sporadic MTC vs. paired normal thyroid tissue (protein)	Zhan et al. (2018)
DBH	Dopamine beta-hydroxylase	Adrenaline and noradrenaline synthesis pathway	P09172	↗ pheochromocytoma vs. non tumor (gene and protein)	Grouzmann et al. (2012), Houy et al. (2022)
DDC	Aromatic-L-amino-acid decarboxylase	Catecholamine metabolic process, dopamine biosynthetic process	P20711	↗ pheochromocytoma vs. non tumor (gene and protein)	Houy et al. (2022)
FARP1	FERM, ARHGEF and pleckstrin domain-containing protein 1	Guanine nucleotide exchange factor for Cdc42 and Rac1, actin cytoskeleton organisation	Q9Y4F1	↘ pheochromocytoma vs. non tumor (gene and protein)	Croise et al. (2016)
MAOA	Amine oxidase [flavin-containing] A	Catecholamine catabolic process	P21397	↘ pheochromocytoma vs. non tumor (gene and protein)	Grouzmann et al. (2012), Houy et al. (2022)
MAOB	Amine oxidase [flavin-containing] B	Catecholamine catabolic process	P27338	↘ pheochromocytoma vs. non tumor (gene and protein)	Grouzmann et al. (2012), Houy et al. (2022)
MYO5A	Unconventional myosin-Va	Vesicle transport, actin filament organization	Q9Y4I1	↗ pheochromocytoma vs. non tumor (protein)	Houy et al. (2022)
PCSK1	Neuroendocrine convertase 1	Peptide hormone processing	P29120	↗ pheochromocytoma vs. non tumor (protein)	Houy et al. (2022)
				↗ functioning corticotroph piNET vs. silent corticotroph piNET (gene)	Zhang et al. (2022)
PCSK1N	ProSAAS	Peptide hormone processing	Q9UHG2	↗ pheochromocytoma vs. non tumor (protein)	Houy et al. (2022)
				↗ sporadic MTC vs. paired normal thyroid tissue (protein)	Zhan et al. (2018)
PCSK2	Neuroendocrine convertase 2	Peptide hormone processing	P16519	↗ pheochromocytoma vs. non tumor (protein)	Houy et al. (2022)
				↗ sporadic MTC vs. paired normal thyroid tissue (protein)	Zhan et al. (2018)
PFN2	Profilin-2	Actin reorganization	P35080	↗ pheochromocytoma vs. non tumor (protein)	Houy et al. (2022)
PLCB2	Phosphoinositide phospholipase C-beta-2	Lipid metabolism: production of DAG and IP3 from PIP2	Q00722	↗ parathyroid adenoma vs. normal parathyroid (protein)	Arya et al. (2019)
PLCB3	Phosphoinositide phospholipase C-beta-3	Lipid metabolism: production of DAG and IP3 from PIP2	Q01970	↗ parathyroid adenoma vs. normal parathyroid (protein)	Arya et al. (2019)
PLCB4	Phosphoinositide phospholipase C-beta-4	Lipid metabolism: production of DAG and IP3 from PIP2	Q15147	↗ pheochromocytoma vs. non tumor (protein)	Unpublished data
				↘ lactotroph pitNET vs. normal cells (gene)	Cui et al. (2021)
RAB27A	Ras-related protein Rab-27A	Interacts with granuphilin to regulate exocytosis, granule maturation, docking, priming	P51159	↗ pheochromocytoma vs. non tumor (protein)	Houy et al. (2022)
				↗ sporadic MTC vs. paired normal thyroid tissue (protein)	Zhan et al. (2018)
				↘ somatotroph invasive pitNET (secreting) vs. non invasive (less secreting) (protein)	Chen et al. (2023)
RAB27B	Ras-related protein Rab-27B	Exosome secretion regulation, multivesicular endosomes docking, regulation of the number and secretion of platelet dense granules, delivery of granules near the exocytic site	O00194	↗ pheochromocytoma vs. non tumor (protein)	Houy et al. (2022)
				↘ somatotroph invasive pitNET (secreting) vs. non invasive (less secreting) (protein)	Chen et al. (2023)
RAB3A	Ras-related protein Rab-3A	Synaptic vesicle maturation, priming, docking, trafficking, recycling	P20336	↗ pheochromocytoma vs. non tumor (protein)	Houy et al. (2022)
				↗ sporadic MTC vs. paired normal thyroid tissue (protein)	Zhan et al. (2018)
				↘ somatotroph invasive pitNET (secreting) vs. non invasive (less secreting) (protein)	Chen et al. (2023)
RAB3B	Ras-related protein Rab-3B	Vesicle trafficking, vesicle biogenesis, regulates vesicle docking, priming, vesicle size, synaptic vesicle cycle	P20337	↗ functioning corticotroph pitNETs vs. silent corticotroph PitNETs (gene)	Zhang et al. (2022)
RAB3C	Ras-related protein Rab-3C	Priming, docking, trafficking, recycling	Q96E17	↗ sporadic MTC vs. paired normal thyroid tissue (protein)	Zhan et al. (2018)
RAB3D	Ras-related protein Rab-3D	Vesicle trafficking, docking	O95716	↗ pheochromocytoma vs. non tumor (protein)	Houy et al. (2022)
SCAMP1	Secretory carrier-associated membrane protein 1	Protein transport, fusion pore dynamics	O15126	↗ pheochromocytoma vs. non tumor (protein)	Houy et al. (2022)
				↗ sporadic MTC vs. paired normal thyroid tissue (protein)	Zhan et al. (2018)
				↗ GH-secreting pitNET vs. respective normal pituitary (protein)	Ribeiro-Oliveira et al. (2008)
SCG2	Secretogranin-2	Granule biogenesis, peptide hormone processing	P13521	↗ pheochromocytoma vs. non tumor (protein)	Houy et al. (2022)
				↗ pheochromocytoma vs. non tumor (protein)	Guillemot et al. (2012)
				↗ sporadic MTC vs. paired normal thyroid tissue (protein)	Zhan et al. (2018)
SCG3	Secretogranin-3	Granule biogenesis, peptide hormone processing	Q8WXD2	↗ pheochromocytoma vs. non tumor (protein)	Houy et al. (2022)
				↗ sporadic MTC vs. paired normal thyroid tissue (protein)	Zhan et al. (2018)
				↗ somatotroph pitNET vs. normal cells (gene)	Cui et al. (2021)
SCG5	Neuroendocrine protein 7B2	Regulation of pituitary hormone secretion, peptide hormone processing	P05408	↗ pheochromocytoma vs. non tumor (protein)	Houy et al. (2022)
				↗ sporadic MTC vs. paired normal thyroid tissue (protein)	Zhan et al. (2018)
				↗ functioning corticotroph piNET vs. silent corticotroph piNET (gene)	Zhang et al. (2022)
				↗ somatotroph pitNET vs. normal cells (gene)	Cui et al. (2021)
SLC18A1	Chromaffin granule amine transporter	Amine transporter, loading of catecholamines in secretory granules	P54219	↗ pheochromocytoma vs. non tumor (protein)	Houy et al. (2022)
SLC18A2	Synaptic vesicular amine transporter	Amine transporter, loading of catecholamines in secretory granules	Q05940	↗ pheochromocytoma vs. non tumor (protein)	Houy et al. (2022)
SNAP25	Synaptosomal-associated protein 25	Vesicle docking, priming, fusion, SNARE binding	P60880	↗ pheochromocytoma vs. non tumor (protein)	Houy et al. (2022)
				↗ sporadic MTC vs. paired normal thyroid tissue (protein)	Zhan et al. (2018)
				↗ somatotroph pitNET vs. normal cells (gene)	Cui et al. (2021)
SMPD1	Sphingomyelin phosphodiesterase	Glycosphingolipid metabolic process	P17405	↗ pheochromocytoma vs. non tumor (protein)	Houy et al. (2022)
STX1A	Syntaxin-1A	Vesicle docking, priming, fusion, SNARE binding, SNARE complex assembly	Q16623	↗ pheochromocytoma vs. non tumor (protein)	Houy et al. (2022)
STX3	Syntaxin-3	Potentially involved in docking of synaptic vesicles at presynaptic active zones, apical receptor involved in membrane fusion of apical vesicles	Q13277	↗ sporadic MTC vs. paired normal thyroid tissue (protein)	Zhan et al. (2018)
STXBP1	Syntaxin-binding protein 1	Vesicle docking, priming, fusion, SNARE binding, SNARE complex assembly	P61764	↗ pheochromocytoma vs. non tumor (protein)	Houy et al. (2022)
SV2A	Synaptic vesicle glycoprotein 2A	Plays a role in the control of regulated secretion in neural and endocrine cells, enhancing selectively low-frequency neurotransmission, positively regulates vesicle fusion	Q7L0J3	↗ sporadic MTC vs. paired normal thyroid tissue (protein)	Zhan et al. (2018)
				↗ somatotroph pitNET vs. normal cells (gene)	Cui et al. (2021)
SYT1	Synaptotagmin-1	Vesicle docking, priming, calcium binding, plasma membrane binding, SNARE binding, vesicle mediated transport	P21579	↗ pheochromocytoma vs. non tumor (protein)	Houy et al. (2022)
				↗ sporadic MTC vs. paired normal thyroid tissue (protein)	Zhan et al. (2018)
				↘ somatotroph invasive pitNET (secreting) vs. non invasive (less secreting) (protein)	Chen et al. (2023)
SYT2	Synaptotagmin-2	Calcium binding, plasma membrane binding, SNARE binding, vesicle-mediated transport, fusion	Q8N9I0	↗ pheochromocytoma vs. non tumor (protein)	Houy et al. (2022)
				↗ sporadic MTC vs. paired normal thyroid tissue (protein)	Zhan et al. (2018)
SYT7	Synaptotagmin-7	Priming, calcium binding, plasma membrane binding, SNARE binding	O43581	↗ pheochromocytoma vs. non tumor (protein)	Houy et al. (2022)
SYTL4	Synaptotagmin-like protein 4	Docking, calcium binding, plasma membrane binding, SNARE binding	Q96C24	↗ pheochromocytoma vs. non tumor (protein)	Houy et al. (2022)
				↘ somatotroph invasive pitNET (secreting) vs. non invasive (less secreting) (protein)	Chen et al. (2023)
TH	Tyrosine Hydroxylase	Dopamine biosynthetic process	P07101	↗ pheochromocytoma vs. non tumor (gene and protein)	Grouzmann et al. (2012), Houy et al. (2022)
VAMP2	Vesicle-associated membrane protein 2	Vesicle docking, priming, fusion, SNARE binding, SNARE complex assembly	P63027	↘ somatotroph invasive pitNET (secreting) vs. non invasive (less secreting) (protein)	Chen et al. (2023)
				↗ functioning corticotroph pitNETs vs. silent corticotroph PitNETs (gene)	Zhang et al. (2022)

Abbreviations: MTC, medullary thyroid carcinoma; pitNET, pituitary neuroendocrine tumor; siNEN, small intestinal neuroendocrine neoplasm.

## 2 Secretory granules biogenesis and maturation

Biogenesis of secretory granules begins at the trans-Golgi network, where prohormones and granin-family proteins (e.g., chromogranins and secretogranins) are selectively sorted and packaged into immature granules (Kim et al., 2006). These granules then undergo a maturation process involving luminal acidification, condensation of their content, proteolytic processing of prohormones, and removal of non-regulated proteins via clathrin-coated vesicles (Tooze et al., 1991; Arvan and Castle, 1998; Kim et al., 2006; Dembla and Becherer, 2021). During this maturation phase, small-molecule hormones such as catecholamines and serotonin are actively transported, from the cytosol into the granules, by vesicular monoamine transporters (VMATs). This uptake is powered by the proton gradients generated by V-ATPases. In parallel, peptide hormones are proteolytically processed within granules by enzymes such as the prohormone convertases PC1/3 and PC2, assisted by the chaperones like secretogranin V (Morvan and Tooze, 2008; Ma et al., 2021). Together, these steps generate mature granules that are competent for stimulus-dependent exocytosis.

The increase in single cell hormone secretion in NETs could arise from the dysregulation of various step of granule biogenesis and maturation. First, it could be the consequence of secretory granules contents overloading. Several studies reported upregulation of mRNAs or proteins involved in hormone synthesis (Table 1). For example, enzymes involved in catecholamines synthesis (Tyrosine Hydroxylase (TH), Dopamine-β-Hydroxylase (DBH), DOPA decarboxylase (DCC)) are overexpressed in pheochromocytoma (Jarrott and Louis, 1977; Isobe et al., 1998; Eisenhofer et al., 2008; Houy et al., 2022). Conversely, reduced expression of enzymes catabolizing catecholamines may further contribute to increase their levels. Accordingly, the monoamine oxydases (MAO-A/B), which deaminate cytosolic catecholamines, are downregulated in pheochromocytoma potentially leading to increased cytoplasmic catecholamine level (Grouzmann et al., 2012; Houy et al., 2022). Finally, expression of transporters such as VMAT1/2 (SLC18A1/A2) responsible for monoamines loading, is also increased (Table 1; Houy et al., 2022). It is, however, currently unknown whether increasing the number of hormone transporter molecules per granule actually leads to a higher hormone content, as there is a physical constraint beyond which additional filling is not possible.

Second, modulating the expression level of prohormone-processing enzymes that convert prohormones into their active forms can also lead to excessive active hormone production in NETs. This is often the case, for example, with neuroendocrine convertases (PCSK-1/2/1N), which are found upregulated in pheochromocytoma (Houy et al., 2022), medullary thyroid carcinoma (Zhan et al., 2018) and pituitary tumor (Cui et al., 2021; Zhang et al., 2022). Interestingly, secretogranin V, a chaperone for prohormone convertase is also overexpressed in various NETs (Table 1).

Third, the overexpression of secretory granule-resident proteins could reflect an increase in the number of secretory granules per cell. Notably, various chromogranin proteins (CHGA/B, SCG2/3; Table 1) or chromogranin-derived peptides, which are key proteins required for granule biogenesis (Kim et al., 2001; Beuret et al., 2004; Elias et al., 2012; Carmon et al., 2020) have also been found to be overexpressed in various NETs (Guillemot et al., 2006; Guerin et al., 2010; Zhan et al., 2018; Kurczyk et al., 2020; Cui et al., 2021; Houy et al., 2022). Whether this reflects an increase of the granin content per granule or an increase of the number of granules per cell or both requires further investigations.

## 3 Docking, priming and fusion

Our work in pheochromocytoma cells demonstrated that both the number of exocytic events and the kinetic of secretion were enhanced suggesting that late phases of exocytosis (docking, priming and/or fusion) could be modulated (Houy et al., 2022). The core machinery controlling docking, priming and fusion involves SNARE (Soluble N-ethylmaleimide sensitive factor attachment protein receptor) proteins, as well as SNARE-regulating proteins. Fusion between the secretory granule and plasma membrane is energetically unfavorable and requires the assembly of a membrane-bridging complex formed by the SNAREs (Jahn et al., 2024). In most neuroendocrine cells, the SNARE complex mediating the regulated exocytosis of LDCVs typically include SNAP25, Syntaxin1, and VAMP2. The number of SNARE complexes formed at the site of vesicle docking is correlated with the likelihood and speed of vesicle fusion (Mohrmann et al., 2010). Therefore, increasing the number of SNARE proteins can lead to more SNARE complexes, potentially enhancing the exocytosis rate. Accordingly, SNAP25 as well as various Syntaxins and VAMPs are upregulated in several NETs (Table 1) (Lu et al., 2008; Zhan et al., 2018; Cui et al., 2021; Houy et al., 2022; Zhang et al., 2022; Chen et al., 2023).

The assembly of the SNARE complex is not calcium sensitive, even though calcium is the main trigger of exocytosis. Synaptotagmins (Syts), a family of transmembrane vesicular proteins, confer Ca^2+^ sensitivity to membrane fusion in neuroendocrine cells by sensing changes in intracellular Ca^2+^ over a wide dynamic range (Pinheiro et al., 2016). Upon Ca^2+^ binding, synaptotagmins promote SNARE-mediated fusion by lowering the energy barrier primarily through the binding of their C2-domains to anionic phospholipids in the target membrane, which induces local positive curvature and membrane apposition to facilitate membrane merging. Among Syt family members, Syt1 and Syt7 have been implicated in neuroendocrine secretion (Schonn et al., 2008; Mohrmann et al., 2013; Bendahmane et al., 2020; Tawfik et al., 2021). Syt1 is a low-affinity, fast-responding isoform that mediate synchronous exocytosis, whereas Syt7 has higher calcium affinity and contributes to sustained or asynchronous release. Interestingly, Syt 1, 2 or 7 are upregulated in pheochromocytoma, medullary thyroid carcinoma and in somatotroph pituitary tumor (Table 1) (Zhan et al., 2018; Houy et al., 2022; Chen et al., 2023).

Altogether these observations suggest that hormone release might be enhanced either by increasing the number of SNARE complexes, or by modulating calcium sensitivity. Alternatively, the efficiency of exocytosis could also be influenced by proteins that tightly regulate the assembly or disassembly of the SNARE complex such as proteins from Sec1/Munc18-1 family, or even by tethering factors such as Rab GTPases that mediate the initial contact between the secretory granule and the plasma membrane (Baker and Hughson, 2016). For example, syntaxin binding protein 1 (STXBP1, commonly named Munc18-1) is overexpressed in pheochromocytoma (Houy et al., 2022), whereas expression level of the GTPases Rab3a-c or Rab27a-b and granuphilin (sytl4) is modified in pheochromocytoma, medullary thyroid carcinoma and in somatotroph or corticotroph pituitary tumor (Table 1), (Zhan et al., 2018; Houy et al., 2022; Zhang et al., 2022; Chen et al., 2023).

## 4 Actin cytoskeleton remodelling

In addition to the essential and minimal core machinery mediating secretory granule recruitment, docking, priming and fusion, an important extra-layer of regulation finely tunes calcium-regulated exocytosis, and consequently, the amount of hormone released. Notably, the functional importance of the actin cytoskeleton in regulated exocytosis has been recognized for several decades. Early studies revealed a dense cortical actin layer beneath the plasma membrane in secretory cells such as pancreatic β-cells and adrenal chromaffin cells (Orci et al., 1972; Trifaro et al., 1985; Aunis and Bader, 1988). These observations initially led to the view that cortical actin served primarily as a physical barrier restricting granule access to fusion sites. Accordingly, numerous studies demonstrated that remodeling of this cortical actin barrier facilitates secretory granules transport and fusion during calcium-regulated exocytosis in a variety of secretory cell models (Koffer et al., 1990; Vitale et al., 1991; Vitale et al., 1995; Gasman et al., 1997; Lang et al., 2000; Giner et al., 2007; Kalwat et al., 2013; Uenishi et al., 2013). Subsequent investigations, also demonstrated that actin filaments not only exert inhibitory control but can also play facilitatory roles, depending on the specific step of secretory granule exocytic process. This evolving concept has been discussed in several comprehensive review articles (Trifaro et al., 2008; Papadopulos, 2017; Li et al., 2018; Wu and Chan, 2022). Therefore, actin reorganization is now recognized as a dynamic and tightly regulated process that both constrains and facilitates exocytosis, depending on context and timing.

Members of the Rho GTPases family regulate exocytosis through actin reorganization. Seminal work from our team has demonstrated that the GTPases RhoA and Cdc42 play key regulatory roles on catecholamine release from adrenal chromaffin cells, by differentially influencing actin organization (Gasman et al., 1999; Malacombe et al., 2006a; Momboisse et al., 2011). RhoA has been proposed to actively control the organization of the cortical actin network, which regulates granule positioning and their access to the plasma membrane. In contrast Cdc42 promotes *de novo* actin nucleation and polymerization at the secretory granule fusion sites, likely controlling the final step of exocytosis such as membrane fusion (Malacombe et al., 2006b; Bretou et al., 2014). Interestingly, we previously showed that Cdc42 activity is inhibited in human pheochromocytoma, suggesting that actin reorganization at the exocytic sites may be affected (Croise et al., 2016; Croise et al., 2017). Although Cdc42 expression remains unchanged compared to non-tumor tissue, we have found that its activity is directly correlated to reduced expression of FARP1, a guanine nucleotide-exchange factor (GEF), that activates Cdc42 (Croise et al., 2016). Moreover, the expression of several GEFs for Rho-GTPases members is modulated in various NETs (for details, see Table 2 in (Streit et al., 2020)). However, whether these changes in Rho-GTPases activity are directly linked to altered hormone release in NETs has not been thoroughly explored.

Annexin-A2 is another important protein that regulates hormone release, and organizes the actin cytoskeleton (Figure 1B). Its expression is altered in various cancers including NETs (Christensen et al., 2018). In adrenal chromaffin cells, annexin-A2 promotes the formation of actin bundles required for efficient secretory granule docking and fusion (Gabel et al., 2015). Changes in annexin-A2 expression have been documented in several NETs, though with some differences. For instance, expression of annexin-A2 is decreased in pheochromocytoma (Houy et al., 2022) and pituitary adenoma (Ribeiro-Oliveira et al., 2008; Cui et al., 2021), but increased in medullary thyroid carcinoma (Kurczyk et al., 2020) and in parathyroid adenoma (Giusti et al., 2011). It would be valuable to study the relationship between the bundling activity of annexin-A2, its expression level and the secretory activity of NET cells. How these actin bundles participate to the exocytic process requires further investigations.

Actin cables may serve to tether, displace secretory granules at the correct location or provide forces to deform membranes. To do so, actin filaments need molecular motors such as myosin proteins (Figure 1B). Myosin II, V and VI constitute the main myosins involved in hormone release by neuroendocrine cells (Papadopulos et al., 2013; Gutierrez and Villanueva, 2018). We found that expression of unconventional myosin-Va is increased in pheochromocytoma (Houy et al., 2022). Interestingly cancer-associated mutations in the MYO5B gene have been described in pheochromocytoma and paraganglioma (Wilzen et al., 2016). Moreover, metastatic pheochromocytoma displays significantly higher expression of Myo5B compared to non-metastatic tumor (Tomic et al., 2020). To our knowledge, no data regarding the expression of Myosins II and VI in human NETs samples has been documented so far.

## 5 Lipid modifying enzymes

Lipids, with their vast diversity encompassing tens of thousands of species, are essential to cellular function, serving structural, metabolic, and signaling roles (van Meer et al., 2008). Early studies demonstrated that the synthesis, metabolism or transport of various lipids species play a fundamental role in hormone secretion by altering membrane biophysical properties and modulating the activity proteins that governs each step of vesicle transport and fusion; for review see (Tanguy et al., 2016; Gasman and Vitale, 2017). Among these lipids, phosphatidylinositol-4,5-bisphosphate (PtdIns(4,5)P_2_) (Martin, 2012), certain fatty acids (Darios et al., 2007), anionic lipid such as phosphatidic acid (PA) (Tanguy et al., 2020), or cholesterol (Lang, 2007) regulate the activity or recruitment at exocytic sites of more than twenty proteins involved in regulated exocytosis (Koch and Holt, 2012). These targets include synaptotagmin (Schiavo et al., 1996), syntaxin (Lam et al., 2008), Ca^2+^-dependent activator protein for secretion (CAPS) (Loyet et al., 1998), and actin-binding proteins such as scinderin and gelsolin (Trifaro et al., 2008).

Although comprehensive lipidomics for NETs are still lacking, transcriptomic and proteomic studies revealed dysregulation of multiple lipid-metabolic enzymes and lipid transporters, whose activity can influence hormone secretion. For instance, phospholipase C enzymes, which hydrolyze (PtdIns(4,5)P_2_) to generate diacylglycerol (DAG) and inositol 3-phosphate, are upregulated in NET subtypes. PLC-β2 and -β3 isoforms are overexpressed in parathyroid adenomas, whereas PLC-β4 expression is elevated in pheochromocytoma and in pituitary NETs, suggesting an imbalance in these lipids in tumor cells (Bauer et al., 2007; Arya et al., 2019; Cui et al., 2021). In pheochromocytoma the sphingomyelin phosphodiesterase, which catalyzes the conversion of sphingomyelin into ceramide and phosphatidylcholine is overexpressed, increasing the pool of phosphatidylcholine potentially available to phospholipase-D1 and thereby potentially augmenting PA-driven exocytosis (Vitale et al., 2001; Zeniou-Meyer et al., 2007; Tanguy et al., 2020).

Hormone secretion is further modulated by the asymmetric distribution of phospholipids across the plasma membrane bilayer. Notably, we have shown that calcium-regulated exocytosis requires a transient disruption of the plasma membrane asymmetry near vesicle fusion sites in adrenal chromaffin cells (Ory et al., 2013). This process is regulated by scramblases, which catalyze the bidirectional transport of phospholipids between plasma membrane leaflets. Interestingly, several transbilayer lipid transporters are overexpressed in pituitary NETs and pheochromocytoma, including the scramblase ANO6 and the P4-type ATPases ATP8A1 (Cui et al., 2021; Houy et al., 2022) and ATP9A (unpublished).

Overall, lipid metabolism and transport appear to be altered in several NETs, which could directly affect hormone secretion. However, further investigations are needed to understand how changes in plasma membrane lipid composition and asymmetry precisely affect exocytosis efficiency.

## 6 Conclusion

Neuroendocrine tumors (NETs) represent a unique class of neoplasms characterized by their aberrant and unregulated hormone secretion, leading to significant clinical consequences. While our understanding of the molecular and cellular mechanisms underlying this hypersecretion is still evolving, recent advances, particularly through “omics” technologies, have begun to shed light on key alterations within the secretory pathway. Current data suggest that disruptions in hormone metabolism and secretory granule trafficking, as well as changes in expression of the regulatory proteins involved in this process, might contribute to the dysfunctional hormone release observed in NETs. Despite this progress, significant gaps in knowledge remain and the next challenge will be to better understand the precise mechanisms by which NET cells acquire such hypersecretory phenotypes. Over the past decade, “omics” technologies have advanced tremendously, enabling extremely fine levels of analysis.

These data, along with the future “omics” research, will be crucial in paving the way for novel therapeutic strategies aimed at targeting the secretory machinery to alleviate the clinical burden of NETs. From a clinical perspective, hormone hypersecretion in NETs is managed through various strategies designed to reduce hormone levels, control tumor growth, and relieve symptoms. Core treatments include somatostatin analogs (SSA), which inhibit hormone release by targeting somatostatin receptors, and peptide receptor radionuclide therapy (PRRT), which delivers targeted radiotherapy to receptor-expressing tumor cells. Surgical resection remains the treatment of choice when feasible, while chemotherapy and targeted agents such as everolimus or sunitinib are used in more advanced stages. Numerous in-depth reviews have covered these therapeutic options extensively (Stueven et al., 2019; Das et al., 2021; Zappi et al., 2023; Faggiano, 2024), including specifically in the NET types discussed in this review, which show significant changes in the expression of proteins controlling exocytosis, such as pheochromocytoma, medullary thyroid carcinoma, and pituitary adenomas (Varlamov et al., 2019; Kim and Kim, 2021; Bihain et al., 2022; Petersenn et al., 2023; Sharma and Fishbein, 2023; Casey et al., 2024). However, these treatments rarely target the secretory pathway itself, and their efficacy can diminish over time. By focusing on the molecular mechanisms of hormone secretion, this review complements the clinical literature and highlights the potential of directly targeting secretory machinery to reduce hormone burden in patients with persistent or refractory symptoms. Finally, despite the availability of several biochemical and imaging biomarkers such as Chromogranin A (CgA), neuron-specific enolase (NSE), and fluorodeoxyglucose positron emission tomography (FDG-PET), the diagnosis of NETs remains challenging. For instance, CgA is the most widely used serum marker, but its limited specificity and frequent false negatives reduce its diagnostic value (Nobels et al., 1997; Nehar et al., 2004). Identifying changes in the expression of secretory granule cargo proteins will certainly help the discovery of new accurate biomarkers.
